# Lower Education and Reading and Writing Habits Are Associated With Poorer Oral Discourse Production in Typical Adults and Older Adults

**DOI:** 10.3389/fpsyg.2022.740337

**Published:** 2022-03-18

**Authors:** Bárbara Luzia Covatti Malcorra, Maximiliano A. Wilson, Lucas Porcello Schilling, Lilian Cristine Hübner

**Affiliations:** ^1^Department of Linguistics, Pontifical Catholic University of Rio Grande do Sul (PUCRS), Porto Alegre, Brazil; ^2^Département de Réadaptation, Faculté de Médecine, Centre Interdisciplinaire de Recherche en Réadaptation et Intégration Sociale (CIRRIS), Université Laval, Quebec, QC, Canada; ^3^Brain Institute of Rio Grande do Sul, Pontifical Catholic University of Rio Grande do Sul (PUCRS), Porto Alegre, Brazil; ^4^National Council for Scientific and Technological Development (CNPq), Brasília, Brazil

**Keywords:** oral discourse, narrative discourse, education, reading and writing habits, typical aging, macrostructure, microstructure, modalization

## Abstract

During normal aging there is a decline in cognitive functions that includes deficits in oral discourse production. A higher level of education and more frequent reading and writing habits (RWH) might delay the onset of the cognitive decline during aging. This study aimed at investigating the effect of education and RWH on oral discourse production in older adults. Picture-based narratives were collected from 117 healthy adults, aged between 51 and 82 years (68.6 ± 6.38) with 0–20 years of formal education (10.1 ± 5.69). Measures of macro, microlinguistic and modalizations were computed and entered as dependent variables in hierarchical regression analyses that included age, education and RWH as regressors. Results revealed that higher education explained a better performance at the macrostructure and microstructure dimensions. Higher frequency of RWH explained the production of fewer modalizations. These results demonstrate the positive effect of education and RWH in oral discourse production in older adults. Therefore, higher attention should be given to these social factors.

## Introduction

Healthy aging involves changes in cognitive functions, including a deficit in oral discourse production (Marini et al., 2005). To generate discourse, the speaker must integrate linguistic and non-linguistic skills to produce and structure a narrative. As a complex task, discourse production is a rich source of information in clinical interviews and cognitive assessment. Thus, discourse production is a valuable tool to support the detection of language and cognitive impairments. Moreover, it is an ecologically valid task, representative of language use in daily life.

During typical aging, discourse comprehension is relatively spared, while discourse production is affected (Ska et al., 2009; Martin et al., 2018). Oral discourse production, the focus of the present study, can be divided into two main dimensions (van Dijk, 2010). The first dimension is the macrostructure. It refers to the semantic information that provides global unity to discourse. The second dimension is the microstructure. It concerns the structure of an individual proposition and its internal relations (van Dijk, 2010). Several studies bring evidence that the ability to deal with the macrostructural dimension of speech decreases more significantly than the microstructural dimension of speech during typical aging. Notably, the oral discourse produced by older adults contains fewer main ideas compared with that produced by younger adults (Glosser and Deser, 1992; Capilouto et al., 2005, 2016; Marini et al., 2005; Wright et al., 2011, 2014). In addition to these two dimensions, meta-discursive strategies, or modalizations, refer to a participant’s comments about the story content or his/her performance during an oral discourse production task (Cardebat et al., 1993; Lira et al., 2018). Studies on modalizations in Alzheimer’s disease (AD) have been developed (Duong et al., 2003, 2005; Toledo et al., 2018), but when it comes to typical aging studies are scarce. For instance, le Dorze and Bédard (1998) compared the use of modalizations by young, middle-aged, and older adults while producing a narrative story based on a single picture (Bank Robbery Picture) (Nespoulous et al., 1986). They observed that older adults included more comments about their difficulties during a narrative task as compared to young and middle-aged adults.

Several factors can delay the onset of cognitive decline during aging. These factors can be grouped under the concept of cognitive reserve. Cognitive reserve establishes that activities that stimulate the brain are linked to an increase in brain resilience to changes in cognitive processing resulting from typical and atypical aging (Stern et al., 2020). Education is one of the factors most associated with cognitive reserve. There is consensus that education is a protective factor that delays the emergence of the cognitive aspects of neurological pathologies (Nitrini et al., 2009; Cotrena et al., 2016; Quintas et al., 2017). In picture-based tasks, studies report better performance in highly educated adults compared with less-educated ones (Ardila and Rosselli, 1996; Duong and Ska, 2001). Few studies addressed the effects of education on the oral discourse production of typical adults. These studies provide evidence that the level of education influences the quantity and completeness of the informational content in tasks based on single pictures, as well as the production of main ideas and cohesive links in tasks based on a sequence of pictures (le Dorze and Bédard, 1998; MacKenzie, 2000; Mackenzie et al., 2007). The present study aims to bring some light to the influence of education in discourse macro and microstructure as well as to modalizations in typical aging.

Another factor associated with cognitive reserve is reading and writing habits (RWH). These habits have recently gained a greater focus of attention regarding their role in typical cognitive aging. A recent study indicates that RWH can contribute to cognitive performance even more than education (Cotrena et al., 2016). Moreover, evidence attests to the positive effects of RWH on executive functions, attention, memory (Pawlowski et al., 2012; Moraes et al., 2013; Sörman et al., 2018), as well as language processing (Pagliarin et al., 2015; Kochhann et al., 2018; Tessaro et al., 2020). For example, Moraes et al. (2013) found that RWH better predicted phonemic fluency than education. Kochhann et al. (2018) found that RWH were the second-best predictor, after verbal fluency, of mild cognitive impairment and AD. Cotrena et al. (2016) found that RWH predicted speed and accuracy in the Hayling test (Burgess and Shallice, 1997), a linguistic measure of executive functions. Sörman et al. (2018) found that the habit of reading books was associated with higher levels of verbal fluency and episodic recall. Tessaro et al. (2020) found that RWH were associated with the total number of words produced in the phonemic verbal fluency task. Pagliarin et al. (2015) found that the combination of education and RWHs better predicted the linguistic performance in oral discourse, in measures such as the number of words, information units (IU), and scenes, rather than one of the variables in isolation.

Overall, these studies corroborate the importance of RWH in cognition. However, to the best of our knowledge, no studies have further investigated the effects of RWH on oral discourse production in typical adults. The effect of education is still scarce as well, as mentioned before. Toward this aim, we used the same picture sequence task and linguistic variables investigated by Lira et al. (2018). Lira et al. (2018) identified the items in the macrolinguistic dimension of oral discourse that better differentiated individuals with AD from typical older adults with a picture sequence task. The present study aims to investigate the effect of education and the frequency of RWH on oral discourse production in typical adults. We hypothesize that higher education levels and a higher frequency of RWH would be associated with better performance on the macro and microstructure dimensions of oral discourse production, as well as with fewer modalizations.

## Method

### Ethical and Data Collection Procedures

The study was approved by the Research Ethics Committee at the university where the study was developed under report number 560.073, CAAE registry number 21006913.0.0000.5336. Participation in the study was voluntary, and participants provided their written informed consent before joining the study. Participants were tested individually in a laboratory setting. The participants were recruited at general courses offered for members of the community at the university and at community centers close to it.

### Participants

One hundred and seventeen adults participated in the study (see Table 1 for their sociodemographic and neuropsychological characteristics). Their ages ranged from 51 to 82 (mean = 68.6; standard deviation, *SD* = 6.38) and education level ranged from 0 to 20 years of formal schooling (mean = 10.1; *SD* = 5.69). They were recruited at community centers, in an urban context in the most southern state in, Brazil. Participants were mainly blue-collar individuals from low to middle-to-low socioeconomic status (SES; see Table 1). The SES scores of the participants in this study ranged from 13 to 41 (mean = 24.2; *SD* = 6.05). The scores for RWHs in the present sample ranged from 0 to 26 (mean = 12.2; *SD* = 6.27). SES was measured with the *Questionário de condição social e questionário de uso de medicamentos*, taken from ABEP—*Associação Brasileira de Empresa e Pesquisa* (2015), which establishes the following cut-off points: lower SES = 0–16; middle = 17–28; upper middle = 29–44; upper = 45–100. RWHs were quantified according to the weekly frequency of reading and writing activities of different types of digital and printed texts. There were four questions for reading habits and four questions for writing habits. Each question had a maximum score of four for a maximum total score of 16 points per modality (reading and writing), thus 32 points in total. The possible scores for each question were as follows: daily (4 points); a few days a week (3 points); once a week (2 points); rarely (1 point), and never (0 points). All participants underwent a battery of linguistic tasks as part of a larger study to map age and dementia-related changes in language processing (BALE–Battery for Language Assessment in Aging) (Hübner et al., 2019). To further characterize the sample, phonological short-term memory and working memory were evaluated with the WAIS-III Digit Span and Backward Digit Span subtests (Wechsler, 1997). Semantic and episodic memories were assessed with two subtests of the BALE. The semantic memory task (naming task) consists of 60 black and white drawings, divided into animate and inanimate and high and low-frequency items, presented in groups of four. The episodic memory task (verbal learning task) consists of identifying and naming 16 figures from different semantic categories and comprised three phases (learning, immediate recall, and 20-min recall) (Table 1).

**TABLE 1:** Descriptive analyses.

Variables	Mean	*SD*	Range	Skewness
Age (in years)	68.6	6.38	51–82	–0.15
MMSE	27.4	2.6	18–30	–1.22
SES	24.2	6.05	13–41	0.62
Education (in years)	10.1	5.69	0–20	–0.03
RWH (min = 0; max = 32)	12.2	6.27	0–26	–0.17
GDS	1.59	1.48	0–5	0.68
Verbal learning (free recall) (min = 0; max = 16)	31.8	7.65	1–45	–1.38
Verbal learning (cued recall) (min = 0; max = 16)	14.9	5.69	3–30	0.37
Verbal learning (late recall) (min = 0; max = 16)	15.6	1.42	7–16	–1.45
Naming (min = 0; max = 60)	54.4	4.04	38–60	–1.34
Digit span forward	7.74	2.11	4–14	0.71
Digit span backward	4.13	1.83	0–9	0.35
Mods	0.186	0.202	0–1.11	1.62
Mps	3.79	2.13	0–6	–0.585
GCoh	6.29	3.17	0–14	–0.29
CCPs	0.666	0.272	0–1	–0.79
NoCCPs	0.25	0.23	0–0.933	1.09
IncPs	0.083	0.112	0–0.556	2.02
CohDs	0.171	0.067	0–0.325	–0.29
Ref	0.058	0.032	0–0.129	0.202
Lex	0.055	0.026	0–0.129	0.362
Conj	0.028	0.018	0–0.82	0.845
Eli	0.026	0.021	0–0.101	0.934
Est	0.002	0.004	0–0.149	1.33
CohEs	0.038	0.036	0–0.171	1.48
Eref	0.008	0.012	0–0.67	2.0
Econj	0.001	0.004	0–0.286	1.87
Einfo	0.016	0.019	0–0.114	1.03
Emissing	0.008	0.012	0–0.508	1.4
Esent	0.004	0.007	0–0.286	1.8
LCoh	10.3	4.72	0–24	0.116

*SD, standard deviation; MMSE, Mini-Mental State Exam, with cut-off points established by Laks et al. (2003); SES, socioeconomic status measured with the Questionário de condição social e questionário de uso de medicamentos, taken from ABEP—Associação Brasileira de Empresa e Pesquisa (Brasil and de, 2015) (lower SES = 0–16; middle = 17–28; upper middle = 29–44; upper = 45–100); RWH, reading and writing habits quantified according to the weekly frequency of reading and writing activities with different types of texts, with ratings classified as: daily (4 points); a few days a week (3 points); once a week (2 points); rarely (1 point), and never (0 points) (Hübner et al., 2019); Depression (Geriatric Depression Scale–GDS, Brazilian version of Almeida and Almeida, 1999); verbal learning task (episodic memory) (BALE) (Hübner et al., 2019); short-term memory and working memory (WAIS-III Digit Span and Backward Digit Span subtest) (Wechsler, 1997); Mods, modalizations; Mps, macropropositions; GCoh, global coherence; CCPs, content-related complete propositions; NoCCPs, no-content-related complete propositions; IncPs, incomplete propositions; CohDs, cohesive devices; Ref, referential; Lex, lexical; Conj, conjunction; Eli, ellipsis; Est, structural; CohEs, cohesive errors; Eref, no reference; Econj, conjunction error; Einfo, information error; Emissing, missing element; Esent, inappropriate sentence; LCoh, local coherence.*

All participants were native Brazilian Portuguese speakers and did not speak other languages. All participants had a general cognitive performance within the normal range as measured by the Mini-Mental State Examination (MMSE; mean = 27.4; *SD* = 2.6) (Laks et al., 2003). We followed the Brazilian scoring procedure provided by Laks et al. (2003). This scoring procedure is adapted for the Brazilian population and takes into consideration age and level of education. Participants did not present with depression, as measured by the Geriatric Depression Scale (GDS) (Yesavage et al., 1982), and did not present any functional problems that attested any cognitive decline indicative of dementia, as measured by the Pfeffer Questionnaire (Pfeffer et al., 1982). As self-reported, they did not present with a current or previous history of neurological disorders, nor a current or previous history of substance abuse, or untreated vision and/or hearing problems.

### Picture Sequence Task

The picture sequence task is known as “The dog story,” a subtest of the Battery for Language Assessment in Aging (Hübner et al., 2019). Participants are asked to tell the story based on seven scenes, following this instruction: “I will show you a story with scenes. Each scene is a moment in the story, which has a beginning, middle, and end. I will ask you to take a good look at the scenes and try to understand the story. Then, I am going to ask you to tell me this story as if you were going to tell it to a friend. Are you ready? Can we start?” The scenes are presented in the correct sequence and remained in front of the participant so that s/he can observe them while telling the story. Participants are given time to observe the scenes and, if necessary, the instruction is repeated. There is no time limit for the accomplishment of the task. The examiner only interferes to encourage the participant if s/he does not show initiative to produce the story. In this case, expressions such as “tell me more,” “can you continue?,” “Uhum,” “and then, what happens?” are addressed. All discourse samples were audio-recorded for later transcription and data scoring. Two separate transcripts were made by two independent researchers (BLCM and LCH) and checked, and disagreements were discussed in consensus.

### Oral Narrative Variables Computation

We used 19 variables. Their explanation can be seen in Table 2. Following Lira et al. (2018), we analyzed one variable for modalizations (Mods), two for macrostructure [macropropositions (Mps) and appropriated global coherence (GCoh)] and 16 for microstructure [content-related complete propositions (CCPs); no-content-related complete propositions (NoCCPs); incomplete propositions (IncPs); cohesive devices (CohDs) and its five subtypes: referential (Ref), lexical (Lex), conjunction (Conj), ellipsis (Eli), or structural (Est); cohesive errors (CohEs) and its five subtypes: no reference (Eref), conjunction error (Econj), information error (Einfo), missing element (Emissing), or inappropriate sentence (Esent); and appropriated local coherence (LCoh)].

**TABLE 2:** Linguistic variables used in the study, based on Lira et al. (2018).

	Variables	Explanation	Example
***Modalizations***			
1	Modalizations (Mods)	The participant’s comments about story content or his/her performance during the task.	*Eu não sei o que é isto/Isto aqui é um cachorro*? (I do not know what this is/Is this a dog?)
***Macrostructure***			
2	Macropropositions (Mps)	The basic components of a narrative structure that summarize the story: (1) a little boy takes a stray dog home; (2) he is worried about his parent’s reaction; (3) he hides the dog in the wardrobe; (4) the mother finds the dog; (5) she asks the boy for an explanation; (6) the mother allows the boy to keep the dog.	–
3	Appropriated global coherence (GCoh)	The frequency of complete or incomplete propositions that are conceptually related to the main topic of the instrument.	*O menino esconde o cachorro no armário*. (The boy hides the dog in the closet.)
***Microstructure***			
4	Content-related complete propositions (CCPs)	The frequency of the propositions with the main predicate and their argument(s) identified in the story.	*O gurizinho viu um cachorro perdido na calçada.* (The boy saw a dog on the sidewalk.)
5	No-content-related complete propositions (NoCCPs)	The frequency of the propositions that present a predicate and their argument(s) but that was not related to the content of the story.	*Uma mulher está atravessando a rua.* (A woman is crossing the street.)
6	Incomplete propositions (IncPs)	The frequency of the propositions lacking a predicate or argument.	*Um menino viu um.* (A boy saw a.)
7	Cohesive devices (CohDs):	The linguistic items used to establish a connection between elements.	–
8	*Referential* (Ref)	An element that presents a semantic relation to a preceding element, such as third-person personal pronouns, possessive pronouns, demonstrative pronouns, or adverb of place.	*A mãe deixa o menino ficar com o cachorro.* ***Ela*** *o ajuda a construir uma casinha*. (The mother lets the boy keep the dog. **She** helps him to build a little house.)
9	*Lexical* (Lex)	The repeated element of a lexical item or the use of a synonym, superordinate, subordinate name, or other semantic related nouns.	*O menino encontra o cachorro e leva o **cãozinho** para casa*. (The boy finds the dog and takes the **little dog** home.)
10	*Conjunction* (Conj)	A word or group of words used to connect clauses with meaningful relationships.	*A mãe aceitou o cachorro e* ***então*** *construiu uma casinha para ele*. (The mother accepted the dog and **then** built him a little house.)
11	*Ellipsis* (Eli)	Elements not emitted due to their redundancy, which refers specifically to preceding sentences or words.	*O menino leva o cachorro para casa e* (***o menino****) esconde ele no armário.* [The boy takes the dog home and (**the boy**) hides it in the closet.]
12	*Structural* (Est)	A non-propositional element that contributes to the continuity of the emitted text, without aggregating meaning.	***Bom***, *o menino está caminhando na rua*. (**Well**, the boy is walking on the street.)
13	Cohesive errors (CohEs):	Elements, present or absent, that disrupt the continuity of meaning in the discourse.	–
14	*No reference* (Eref)	A referring item is present, but the item to which it refers is not specified or evident from the immediate context.	*O menino e o cachorro.* ***Ele*** *vai para casa*. (The boy and the dog. **He** goes home.)
15	*Conjunction error* (Econj)	The use of an inappropriate conjunctive element.	*Aqui é o menino* ***para*** *falar com a moça*. (Here is the boy **to** talk to the girl.)
16	*Information error* (Einfo)	An element that causes a misstatement of the story content.	*O menino está dando comida para o cachorro*. (The boy is feeding the dog.)
17	*Missing element* (Emissing)	An absent element that causes errors in cohesion between words, clauses, or propositions.	*Aqui o menino está* (***elemento faltante***). [Here the boy is (**missing element**).]
18	*Inappropriate sentence* (Esent)	The omission or misuse of an element that contributes to maintaining the grammar structure of the discourse, mainly the verbal or nominal concordance.	***Os menino*** *construíram uma casinha para o cachorro*. (**The boys** built a little house to the dog.)
19	Appropriated local coherence (LCoh)	The frequency of complete or incomplete propositions that are conceptually related to the immediately previous proposition.	*O menino viu o cachorro e o levou para casa*. (The boy saw the dog and took it home.)

*Bold words represent the target linguistic item for each variable of interest.*

Following Lira et al. (2018), we analyzed CCPs, NoCCPs, IncPs, Mps, and Mods as a proportion of all the propositions. The total of the CohDs and CohEs, as well as their subtypes, represent the ratio of the sum of the words produced. GCoh and LCoh were considered as absolute numbers.

### Data Analysis

We analyzed the data using the Tidyverse package (Wickham et al., 2019), implemented in RStudio (R Core Team, 2020).^1^ First, we examined the data for skewness and kurtosis. The values were within the acceptable respective ranges (−2 to +2 for skewness and −9 to +9 for kurtosis) (Schmider et al., 2010), and thus, no transformations were performed (see Table 1). In our regression models, we entered one dependent variable per construct of interest (macrostructure, microstructure, and modalizations). Since macrostructure and microstructure were composed of several variables, we computed two composite scores, one for microstructure and one for macrostructure. For some variables, a higher score indicates better performance; for other variables, a higher score denotes worse performance. For instance, a better score in global coherence indicates better performance, while more cohesive errors indicate a worse performance Thus, we rendered all the variables in the same direction (i.e., higher scores associated with better performance). To that end, we subtracted each individual score from its maximum number. In this way, a higher score always means better performance. Then, we calculated two composite scores by summing the scores of the variables related to the macrostrucuture (Mps, GCoh) and the microstructure (CCPs, NoCCPs, IncPs, CohDs, Ref, Lex, Conj, Eli, Est, CohEs, Eref, Econj, Einfo, Emissing, Esent, and LCoh). Next, we ran a single hierarchical regression model for each one of the constructs of interest (macrostructure, microstructure, and modalizations). This method allows examining the variation in the dependent variable with each subsequent addition of an independent variable (Schmider et al., 2010). We grouped the independent variables in the regression models in three separate steps. Step 1 included age and status socioeconomic, step 2 included education, and step 3 included frequency of RWHs. We examined variance inflation factor (VIF) and Tolerance to assess for multicollinearity. The reference value for the VIF was <4, and for the tolerance, the reference value was >02. The values for VIF and Tolerance were within the acceptable ranges, and thus, there was no multicollinearity issue in this analysis. Model improvement was evaluated using Δ*F*-statistic. Improvement in the explained variance was calculated using Δ*R*^2^. Statistical significance level was assumed at *p* < 0.05. All the data are available in Supplementary Table 1.

## Results

Table 1 provides the means, standard deviations, ranges (minimum and maximal values), and skewness for the demographic and neuropsychological variables of the sample, as well as for all the linguistic measures (dependent variables).

Results of the hierarchical regression analyses, including values of change in *R*^2^ (Δ*R*^2^) and standardized coefficients (β) for the predictor variables at each step are presented in Table 3.

**TABLE 3:** Standardized βs, *R*^2^s, and Δ*R*^2^ for the three hierarchical regression analyses (macrostructure, microstructure, and modalizations).

	Dependent variables		
	
	Macrostructure	Microstructure	Modalizations
**Step 1**			
Age	–0.083	0.026	0.202
Socioeconomic status	0.234*	0.195	–0.017
*R* ^2^	0.068	0.037	0.042
**Step 2**			
Education	0.371***	0.315**	–0.186
*R* ^2^	0.167	0.108	0.067
Δ*R*^2^	0.099***	0.071**	0.024
**Step 3**			
Reading and writing habits	–0.019	–0.024	−0.252*
*R* ^2^	0.167	0.109	0.106
Δ*R*^2^	<0.001	<0.001	0.038*

*Columns refer to the different regression models and their titles show the dependent variable of the model.*

*ΔR^2^ is the incremental increase in the model R^2^ that results from the addition of a predictor or set of predictors in a new step of the model.*

*p < 0.05, **p < 0.01, and ***p < 0.001.

### Modalizations

**FIGURE 1:**
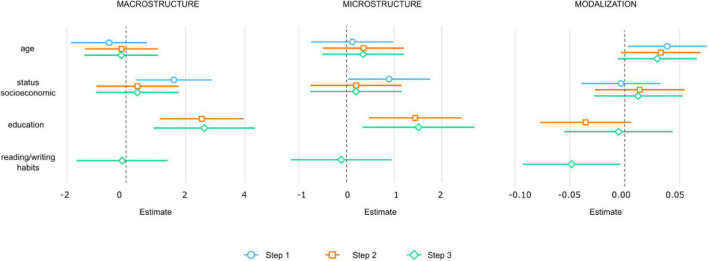
Beta and confidence intervals for each regressor variable of the three hierarchical linear regression models (macrostructure, microstructure, and modalizations). The dots represent the beta, and the lines depict the confidence intervals for each regressor variable of the models.

In the first step of the regression analysis, neither age nor socioeconomic status significantly predicted modalizations [*R*^2^ = 0.042, *F*_(2, 109)_ = 2.409, *p* = 0.094], and the addition of education in Step 2 did not accounted for a significant increase in the variance of modalizations beyond of that explained by the previous sets of predictors [*R*^2^ = 0.067, Δ*R*^2^ = 0.024, *F*_(3, 108)_ = 2.595, *p* = 0.056]. However, in Step 3, RWH accounted for a significant amount of variance in modalizations [*R*^2^ = 0.106, Δ*R*^2^ = 0.038, *F*_(4, 107)_ = 3.179, *p* = 0.016] beyond the variance explained by the variables entered in the two previous steps. RWH significantly contributed to the change in variance in Mods [β = −0.252, *t*(107) = −2.160, *p* = 0.033], indicating that the higher the frequency of RWH, the fewer the comments on the content of the story or on the participant’s own performance during the task (modalizations) (Figure 1).

### Macrostructure

In the first step of the analysis, socioeconomic status accounted for a significant amount of variance in macrostructure score [*R*^2^ = 0.068, *F*_(2, 109)_ = 3.99, *p* = 0.021]. Socioeconomic status was found to significantly contribute to the change in variance in macrostructure [β = 0.234, *t*(109) = 2.505, *p* = 0.013], indicating that the higher the socioeconomic status, the better the performance at the macrostructure level. The addition of education in the step 2 accounted for a significant increase in the variance of macrostructure [*R*^2^ = 0.167, Δ*R*^2^ = 0.099, *F*_(3, 108)_ = 7.234, *p* ≤ 0.001] beyond that explained by the previous sets of predictors. Education significantly contributed to the change in variance in macrostructure [β = 0.371, *t*(108) = 3.585, *p* ≤ 0.001]. In step 3, the addition of RWH did not accounted for a significant amount of variance in macrostructure score [*R*^2^ = 0.067, Δ*R*^2^ ≤ 0.001, *F*_(4, 107)_ = 5.384, *p* ≤ 0.001] (Figure 1).

### Microstructure

In the first step of the regression analysis, neither age nor socioeconomic status significantly predicted microstructure score [*R*^2^ = 0.037, *F*_(2, 109)_ = 2.112, *p* = 0.125]. However, the addition of education in step 2 accounted for a significant increase in the variance of microstructure beyond that explained by the previous sets of predictors [*R*^2^ = 0.108, Δ*R*^2^ = 0.071, *F*_(3, 108)_ = 4.397, *p* = 0.005]. Education significantly contributed to the change in the microstructure level [β = 0.315, *t*(108) = 2.944, *p* = 0.003], indicating that the higher the education, the better their performance at the microstructure level. In step 3, the addition of RWH did not account for a significant amount of variance in microstructure score (Figure 1).

## Discussion

This study aimed at investigating the effects of education and RWH on oral discourse production in typical adults. These factors are known to increase cognitive reserve during aging (Stern et al., 2020). We hypothesized that higher education levels and frequency of RWH would be positively associated with better performance on the macro- and microstructure dimensions of oral discourse production, as well as with fewer modalizations. Our results indicate that higher education explained the production of higher macro and microstructure scores. Moreover, our results indicate that higher frequency of RWH explained the production of fewer modalizations.

While the study developed by Lira et al. (2018) showed significant differences between AD participants and healthy controls regarding the macro- and microstructure dimensions, mainly macropropositions, global coherence, and the ellipsis subtype of cohesive devices [regarding modalizations, Lira et al. (2018) did not find differences between AD and healthy controls groups], our results demonstrate variations in oral discourse production as a function of education and frequency of RWH present in typical adults. Taken together, our results corroborate previous studies that support the positive effects of education (Ardila and Rosselli, 1996; Juncos-Rabadán, 1996; le Dorze and Bédard, 1998; Ardila et al., 2000; MacKenzie, 2000; Duong and Ska, 2001; Bennett et al., 2003; Hong et al., 2011; Nogueira et al., 2016) and frequency of RWH (Pawlowski et al., 2012; Moraes et al., 2013; Cotrena et al., 2016; Kochhann et al., 2018; Sörman et al., 2018; Tessaro et al., 2020) on human cognition. Our findings are discussed below in terms of (a) modalizations, (b) macrostructure, and (c) microstructure.

### Modalizations

Our results showed that as the frequency of RWH increases, the number of modalizations—participants’ comments about their performance during the task—decreases. Studies investigating modalizations in AD patients found that they produced a higher amount of modalizations than healthy participants [Duong et al., 2003; Toledo et al., 2018; but see Cardebat et al., 1993; Lira et al., 2018 for contrasting results]. Regarding typical adults and older adults, le Dorze and Bédard (1998) found that older adults made more comments about their difficulties during a picture description task as compared to younger adults. To the best of our knowledge, the present study is the first one to address the variations in modalizations in oral discourse production as a function of the frequency of RWH in typical adults and older adults. It is possible that individuals who maintain a high frequency of RWH have less difficulty in understanding the story and, therefore, are less susceptible to distractions and to the production of irrelevant information.

Indeed, modalizations can be interpreted as a rupture in the discursive macrostructure, since when individuals discuss their performance they tend to deviate from the theme of the narrative (Cardebat et al., 1993; Lira et al., 2018). In addition, from a pragmatic point of view, the presence of modalizations might reflect the awareness of their difficulties to maintain the central theme of the discourse (Lira et al., 2018; Toledo et al., 2018).

In our study, RWH contributed to explaining variation in modalizations, but did not explain variation at the macro and microstructure levels. Indeed, in previous studies with typical adult and older adult populations, the habit of reading books was associated with higher levels of verbal fluency (Sörman et al., 2018) and the frequency of RWH was associated with the total number of words produced in the phonemic verbal fluency task (Tessaro et al., 2020). Based on this evidence, it could be possible to postulate that individuals who maintain a frequency of RWH in their lives may have a richer vocabulary. Further studies need to address the issue of the relationship between the frequency of RWH and discourse production, which is a complex linguistic ability that goes beyond the word level. Besides, future studies could analyze the impact of RWH in other discourse tasks, as well as analyze picture sequences, like in our study, by adopting automatic language analysis, such as speech connectedness (Mota et al., 2018), to bring more conclusive evidence on the role of frequency of RWH at the discourse level.

### Macrostructure

According to van Dijk (2010), the macrostructural dimension refers to the most relevant or prominent topic in the semantic information of the discourse. Our results reveal that as education increases, the performance at the macrostructure dimension increases as well. This finding corroborates other studies, which indicated that individuals with a higher level of education present more informative content in their narratives and better recognize and use the structure of the story than individuals with a lower level of education (Juncos-Rabadán, 1996; le Dorze and Bédard, 1998).

Many studies presented in the literature indicate that global coherence is the most affected aspect of oral discourse production in people with AD (Glosser and Deser, 1991; Chapman et al., 1995; Laine et al., 1998; Dijkstra et al., 2002, 2004; Ash et al., 2007; Brandão et al., 2013; Lima et al., 2014; Drummond et al., 2015; Lira et al., 2018; Toledo et al., 2018; Pistono et al., 2019). Our results indicate that coherence also plays an important role in typical aging. Moreover, our results corroborate the findings of Mackenzie (2000), who also found that individuals with higher education levels performed better than individuals with lower educational levels on the macrostructure dimension.

### Microstructure

According to van Dijk (2010), the microstructural dimension of the discourse refers to the structure of an individual proposition and its internal relations. Our results indicate that as education increases, the performance at the microstructure dimension increases as well. Mackenzie et al. (2007) found that participants with lower educational levels produced more tangential sentences in their narratives than participants with higher educational levels. In another study, Mackenzie (2000) found that participants with a lower level of education produced shorter and less complete narratives in comparison with the more educated participants. Other studies of cohesion in healthy adults and older adults have reported similar findings (Juncos-Rabadán, 1996; le Dorze and Bédard, 1998). Juncos-Rabadán (1996) found that older adults with a higher level of education used more cohesive links in their narratives, while older adults with a lower level of education used a greater number of descriptive sentences and deictic elements in their narratives. These results may indicate that picture-based tasks are sensitive to the level of education and might also provide a sensitive indicator of the linguistic competence of healthy adults. In such tasks, participants are limited to the content of the pictures and cannot resort to compensatory strategies as, for example, in a rehearsed autobiography of a family narrative (Wright et al., 2011). Overall, these results stress the importance of education in the microstructural dimension of the discourse.

It is important to note that age did not predict any of the dependent variables, unlike other similar studies that addressed this effect, such as Capilouto et al.’s (2016). However, Capilouto, Wright, and Maddy used a single picture description task. Another difference between our study and Capilouto, Wright, and Maddy’s study that could explain the contrasting results is the age range of participants. Capilouto et al. (2016) divided their participants into three groups according to their age, ranging from 20 to 89. In our study, age ranged from 51 to 82 years old.

The fact that age did not predict any of the linguistic measures may indicate that the most important factors for oral discourse production are social factors and not the age when considering adult and older adult populations. Previous studies have revealed that, as age increases, there are discursive gains. Marini et al. (2005) found that the productions of participants aged 40–74 years presented more main ideas in the task based on a single figure than the productions of participants aged 20–39 and between 75 and 84 years. This can be interpreted as an improvement in the narrative capacity related to aging, which decreases only in older people. Thus, middle-aged groups may have discursive gains, and education plays one of the most important roles in discourse production.

Considering that the present study aims to investigate language at the level of individual differences, one limitation of the study is that it does not consider the overall intellectual or any other measure of general functioning. Therefore, future studies should analyze the impact of general intelligence together with social variables in the oral discourse production of older adults.

In sum, our results demonstrate the positive effect of education on the macro- and microlinguistic aspects of oral discourse production during typical aging, as well as the positive effects of frequently engaging in RWHs. Moreover, the use of narrative tasks based on a sequence of pictures seems to be valid to detect differences in oral production between healthy adult and older adult populations regarding their schooling and RWHs, showing its efficiency as a tool to be used in the clinics and in research. Oral narrative productions represent an ecologically valid way to elucidate discourse, which goes beyond the word and sentence levels, therefore favoring the analyses of coherence, cohesion, together with other aspects at the micro- and macrostructural levels, present in daily conversations. Finally, based on our results, which showed the impact of education and RWHs on oral narrative production in healthy adulthood and aging, greater attention should be paid to education and RWHs, since these can prevent or delay the development of neurodegenerative diseases, such as AD. This is especially relevant in underdeveloped or developing countries, where the increase of dementia in the near future is associated with low socioeconomic status and low educational level.

## Data Availability Statement

The original contributions presented in the study are included in the article/Supplementary Material, further inquiries can be directed to the corresponding author.

## Ethics Statement

The studies involving human participants were reviewed and approved by the PUCRS University Research Ethics Committee (report number 560.073, CAAE registry number 21006913.0.0000.5336). The patients/participants provided their written informed consent to participate in this study.

## Author Contributions

BM contributions to included analysis, interpretation of the data, writing, and drafting the submitted material. MW supervised the data analyses and performed a critical revision of the manuscript. LS performed a critical revision of the manuscript. LH supervised the data collection and performed a critical revision of the manuscript. All authors contributed to the article and approved the submitted version.

## Conflict of Interest

The authors declare that the research was conducted in the absence of any commercial or financial relationships that could be construed as a potential conflict of interest.

## Publisher’s Note

All claims expressed in this article are solely those of the authors and do not necessarily represent those of their affiliated organizations, or those of the publisher, the editors and the reviewers. Any product that may be evaluated in this article, or claim that may be made by its manufacturer, is not guaranteed or endorsed by the publisher.

## Footnotes

1
https://www.R-project.org/
